# The Role of Anatomic Connectivity in Inhibitory Control Revealed by Combining Connectome-based Lesion-symptom Mapping with Event-related Potentials

**DOI:** 10.1007/s10548-024-01057-z

**Published:** 2024-06-10

**Authors:** Alex S. T. Nono, Marco Anziano, Michael Mouthon, Joelle N. Chabwine, Lucas Spierer

**Affiliations:** 1 Laboratory for Neurorehabilitation Science, Medicine Section, Faculty of Science and Medicine, University of Fribourg, PER 09, Chemin du Musée 5, 1700 Fribourg, Switzerland; 2 Neurology Unit, Department of Internal Medicine and Specialties, Fribourg Hospital, Fribourg, Switzerland

**Keywords:** Inhibitory Control, Stroke, Event-related Potentials, Lesion Symptom Mapping

## Abstract

Inhibitory control refers to the ability to suppress cognitive or motor processes. Current neurocognitive models indicate that this function mainly involves the anterior cingulate cortex and the inferior frontal cortex. However, how the communication between these areas influence inhibitory control performance and their functional response remains unknown. We addressed this question by injecting behavioral and electrophysiological markers of inhibitory control recorded during a Go/NoGo task as the ‘symptoms’ in a connectome-based lesion-symptom mapping approach in a sample of 96 first unilateral stroke patients. This approach enables us to identify the white matter tracts whose disruption by the lesions causally influences brain functional activity during inhibitory control. We found a central role of left frontotemporal and frontobasal intrahemispheric connections, as well as of the connections between the left temporoparietal and right temporal areas in inhibitory control performance. We also found that connections between the left temporal and right superior parietal areas modulate the conflict-related N2 event-related potential component and between the left temporal parietal area and right temporal and occipital areas for the inhibition P3 component. Our study supports the role of a distributed bilateral network in inhibitory control and reveals that combining lesion-symptom mapping approaches with functional indices of cognitive processes could shed new light on post-stroke functional reorganization. It may further help to refine the interpretation of classical electrophysiological markers of executive control in stroke patients.

## Introduction

Inhibitory control (IC) is a key executive component referring to the ability to suppress cognitive or motor processes (Aron 2007). IC is typically assessed with Go/NoGo tasks in which participants have to respond as fast as possible to a category of stimuli while withholding their responses to other stimuli. Converging neuroimaging and clinical literature indicates that inhibitory control depends on the multi-phase involvement of a cortico-subcortical network comprising the anterior cingulate cortex, the inferior frontal gyrus (IFG), the presupplementary motor area (preSMA) and basal ganglia (BG; for review: Chambers et al. 2009).

After the occipito-temporo-parietal processing of the stimuli and its interfacing with response rules (Mechelli et al. 2004), stimulus-driven demand for inhibitory control is processed by the anterior cingulate cortex, which detects the conflict between the task requirement for motor inhibition and participants’ response tendency (Nieuwenhuis et al. 2003; Sehlmeyer et al. 2010; Wessel et al. 2012). This processing step is typically indexed by the fronto-central N2 event-related potential (ERP) component manifesting around 200–300 ms post stimulus-onset (Veen and Carter 2002; Yeung et al. 2004; Huster et al. 2013). The processing of stop signals then involves the inferior frontal cortices and/or the PreSMA, which generate the inhibition via the subthalamic nucleus (STN) to eventually suppress the final stages of the Go processes up to primary motor cortices (Spierer et al. 2013). The engagement of the fronto-central and ventrolateral prefrontal cortices to inhibit motor action is indexed by the P3 frontal ERP component around 300–500 ms (Kok et al. 2004; Ramautar et al. 2004).

Lesion studies only partly corroborate this functional literature by indicating a decrease in IC performance after damages to the fronto-parietal IC functional network. For instance, Aron et al. (2003) found correlations between the proportion of damage to the anterior cingulate cortex and lateral prefrontal cortex areas with stop-signal reaction time (SSRT), an index of IC performance. In contrast, Picton et al. (2007) found that lesions to the superior medial frontal lobes reduce inhibition accuracy while lesions to the right anterior cingulate cortex were associated with impairment in error monitoring.

Yet, while accumulating evidence indicates a key role of anterior-cingulate and inferior frontal structures, whether and how the connections within these nodes and between this network and the perceptual-attentional areas are involved in performance and in their functional responses remains unclear. A role of these anatomic connections could however be hypothesized as they would support the integration and coordination of the sequence of cognitive processes necessary for an optimal IC (Swick et al. 2011).

We addressed this question by injecting the amplitudes of the N2 and P3 components of ERP measured during a classical Go/NoGo inhibitory control task as the ‘symptom’ in connectome-based-lesion-symptom-mapping analyses [CLSM; (Gleichgerrcht et al. 2017; Griffis et al. 2021)]. This approach uses reference tractography and gray matter parcellations atlases to estimate the structural disconnection caused by lesions in individual patients. Statistical analyses examining the relationship between these models and electrophysiological components of IC are then conducted to identify how the severity of white matter (WM) lesions and the degree of disconnection between distant brain regions influence the symptoms of interest.

We posit that lesions to the tracts connecting the core IC areas (rIFG, pre-SMA, and BG) will result in impaired motor inhibitory control, together with reductions in the amplitude of the N2 and P3 components. To our knowledge, our study is the first to combine lesion-symptom mapping approaches with functional indices of cognitive processes. Such an approach could not only provide unprecedented insights into the functional organization of the damaged brain, but also allow for a more complete exploitation of functional data in stroke patients.

## Method

### Participants

We recruited patients from the Fribourg Hospital who had a first unilateral stroke between 2017 and 2022. All procedures were approved by the local ethics committee (protocol 2016–00517). Each patient provided a written informed consent to participate in the study and was allowed to withdraw their participation any time without needing to provide explanations.

The inclusion criteria for participating in this study were: i) first unilateral hemispheric lesion documented by MRI, CT scan and/or radiological report; ii) age ≤ 85 years. Exclusion criteria were: i) antecedents of neurologic or psychiatric disorders; ii) cerebellar, bihemispheric stroke, or subarachnoid hemorrhage; and iii) difficulty understanding the task.

Our minimal sample size was identified as being 80, based on previous literature stating the sufficient dimension of the database to obtain sufficient brain coverage and analytical power in lesion-symptom mapping (Kimberg et al. 2007) and on the availability of clinical data. One hundred and four patients were recruited for the study, but eight patients were excluded from the analysis due to incomplete data and technical errors during the recording process. A total of ninety-six patients (mean age ± SD = 65.1 ± 10.1) were thus eventually included in the data analyses. We assigned the values 1, 2, and 3, respectively, to patients based on distinct levels of education: those with less than 10 years of education, those with between 10 and 15 years of education, and patients with more than 15 years of education (Table 1).

**Table 1:** Detailed demographic information

	Number or range	Mean	SD	Median
Participants	96	-	-	-
Age (years)	41—85	65.1	10.1	65
Gender (M/F)	80/16	-	-	-
Education (1–2-3)	1–3	2.15	0.7	2
Etiology (ischemic/hemorrhagic)	90/6	-	-	-
Lesion side (R/L)	28/68	-	-	-
Lesion size (% of total brain volume)	0.001–1.72	0.2	0.33	0.04
Stroke- Go/NoGo interval (days)	60–219	136	33.7	143

### Stimuli

We used the same visual Go/NoGo task as in our previous studies (De Pretto et al. 2016; Hartmann et al. 2016). Visual stimuli were colored letters (blue, cyan, green, red, white or yellow ‘A’, ‘E’, ‘M’, ‘O’, ‘S’ or ‘T’) presented in the center of a black screen. Each possible combination of the letter and color was used, for a total of 36 different stimuli. In a given block, NoGo stimuli were either all letters of a given color or all colors of a given letter (total 12 different NoGo stimuli); Go trials were all the remaining stimuli. Go and NoGo stimuli were equally probable.

### Procedure and Task

The procedure and task were the same as in our previous study Hartmann et al. (2016). We will thus report only the essentials here. Patients completed a Go/NoGo task in which they were instructed to respond as fast as possible to Go stimuli by pressing a button on a response box with their index finger while withholding their responses to NoGo stimuli. Stimulus presentation and response recording were controlled by the E-Prime 2.0 software (Psychology Software Tools, Inc., Sharpsburg, PA).

There were 8 blocks of 60 trials separated by 3 min breaks. Each block consisted of 60 trials: 30 Go and 30 NoGo trials presented randomly. The NoGo stimuli (i.e., a given color or letter) were pseudorandomly determined for each block and across patients, so that there was never two times the same NoGo for a given patient and that the order of the NoGo was different for each patient.

Before the beginning of each block, patients were told which stimuli were the NoGo for the block. Patients then completed a calibration block of 12 trials (6 Go; 6 NoGo) during which the mean response time (RT) to Go trials was calculated. This mean RT (RT threshold, RTt) was then used as a threshold during the following experimental block: if the RT to a Go trial was greater than 90% of the mean RTt, a feedback ‘Too late!’ was presented at the end of the trial. This procedure enabled maintaining the same level of time pressure across patients and blocks, i.e., independent of any initial inter-individual differences in Go/NoGo performance (for similar procedures: Vocat et al. 2008; Manuel et al. 2010). No feedback was given on performance during the calibration block and patients were kept naive to the aim of the calibration block.

**Fig. 1:**
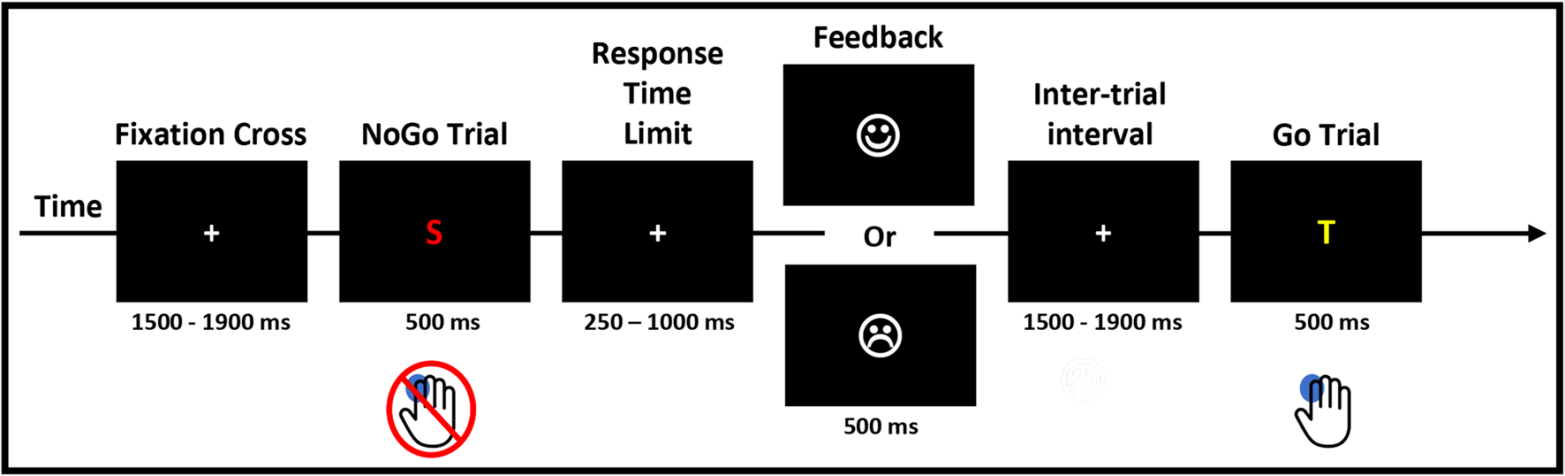
Experimental visual Go/NoGo paradigm. Participants had to respond as fast as possible to the Go stimuli while withholding their response to the NoGo stimuli. A feedback was displayed on response speed and accuracy

Each trial started with the presentation of a gray fixation cross during a time range of 1500–1900 ms, followed by the stimuli (500 ms) and a response window (Fig. 1). Independent of the response time threshold determined during the calibration phase, the response window terminated as soon as the participant responded, but had a minimal and a maximal duration of 250 and 1000 ms, respectively. Then, the participants received a feedback on their performance for 500 ms: a happy smiley icon after Hits (response after a Go stimulus); a feedback “Too late!” replaced the happy smiley after hits with a RT > RTt; a happy smiley after correct rejections (no response after a NoGo stimulus); and an unhappy smiley after misses (no response after a Go stimulus) or false alarms (response after a NoGo trial).

### Behavioral Analysis

Two measures of behavioral performance were calculated from the Go/NoGo task across all trials of a session: the average RT to Go stimuli with correct responses (“Hit” trials) and the percentage of errors in NoGo trials (false alarms, FA rate). Based on these two indices, we computed the performance index, calculated as 100 minus the percentage of FA divided by the response time to the Go stimuli, where higher values indicate better performance. This index has the advantage of considering both the speed and accuracy, and thus to provides a global index of performance insensitive to speed-accuracy tradeoffs. Compensatory strategies after brain lesions may indeed lead to the development of speed- vs accuracy- based response strategy, which may be missed by focusing only on one of the metrics to index performance.

Sensitivity (d') was calculated according to the signal detection theory (SDT)(Green and Swets 1966), as the difference between z-transformed hit rate to Go items and z-transformed false alarm rate to NoGo items. Due to extreme Hit and FA proportions, we adopted the Hautus’ log-linear correction method, which adds 0.5 to all cells in the contingency table regardless of the cell’s contents to calculate the Hit and FA rate (Hautus 1995). This analysis was performed using the dprime function in the “psycho” R package (Makowski 2018).

### EEG Recording

The 64-channel electroencephalogram (EEG) was recorded at a sampling rate of 1024 Hz with a Biosemi ActiveTwo system (Biosemi, Amsterdam, Netherlands) referenced to the common mode sense-driven right leg (CMS-DRL). Offline preprocessing and statistical analyses of the ERPs were conducted using custom MATLAB script relying on EEGLab and the cartool freeware programmed by Denis Brunet (brainmapping.unige.ch/cartool).

### EEG Analyses

#### Event-related Potentials Preprocessing

For each participant, we extracted and averaged EEG epochs for successful NoGo stimuli (correct rejections). The preprocessing of the raw data was conducted using the same methods as in our previous study (Wicht et al. 2022), specifically:

- Re-referencing to Cz electrode and band-pass filtering (0.5 – 40 Hz);
- Artifacts removal on continuous data with the EEGLab plugins, i) CleanLine (sinusoidal, line noise frequencies removed: 50/100 Hz (*see* (Mullen 2012); ii) Artifact Subspace Reconstruction (ASR: non-stationary signals > 10SD from mixing matrix calculated on a clean “reference” section of the recording; *see* Mullen et al. 2015; Chang et al. 2018). The average number of trials across subjects after artifact removal of 193 ± 28.4;
- Epochs' segmentation time-locked to stimulus onset (100 ms pre-to 700 ms post-stimulus onset);
- Baseline correction on the whole epoch window was performed to the interval from 0 to 102 Time frame;
- Interpolation of bad channel(s) using multiquadric interpolation relying on radial basis functions (*see* (Buhmann 2000; Jäger et al. 2016; Jäger 2018). Electrodes were selected based on identification from the averaged ERPs. After interpolation, the average percentage of interpolated channels across subjects was 1.51 ± 2.10%;
- Epoch averaging for each patient across the condition of correct rejections condition (CR, correctly not responded NoGo trials);
- Re-referencing to common average reference.

#### Event-related Potentials Analyses

After ERP pre-processing, the period of interest (POI) for condition-level analyses was determined based on the N2 and P3 ERP components on the condition-averaged Global Field Power (GFP) waveform. The GFP is a measure of the strength of electrical field potentials, calculated as the standard deviation of the mean voltage amplitude over all electrodes at a given time point (Michel and Murray 2012). The GFP peak during the component-specific POI represents the time point when each component exhibits maximum neuronal synchronization, resulting in the highest signal-to-noise ratio (Michel and Murray 2012). To determine each component, the latency and topography of the GFP peaks on the grand average of all subjects were examined. Specifically, a GFP peak around 300 ms with fronto-central negativity for the Nogo-N2, and around 400 ms with fronto-central and central positivity for the Nogo-P3 components, respectively. Once each component was identified, the POI was defined as the component peak latency of the mean GFP ± 40 ms from the peaks of the grand average GFP. Subsequently, individual subjects’ component amplitudes were calculated by averaging their individual GFP voltage over the POI for each component of interest. To conduct these analyses and to determine the POI, we used scripts from our previous studies (available at the following address: https://github.com/CorentinWicht/GFPPeaks (Wicht 2020)). Analyzing the GFP provides an interpretative advantage over analyses of local ERP waveforms, as it considers the entire electrode montage, is reference-independent, and is insensitive to spatial (i.e., topographic) changes in ERP.

### Image Processing

We collected structural CT/MRI brain images (4 CT/92 MRI) of patients, acquired as part of the routine clinical stroke investigations, from the database of the Cantonal Hospital of Fribourg, Switzerland.

For creating lesion masks, two different methods were used depending on the quality of the original brain images. The high-quality images were processed semi-automatically using SPM12 (SPM12—Statistical Parametric Mapping. Alternate URLs. http://www.Nitrc.Org/Projects/Spm, https://www.Fil.Ion.Ucl.Ac.Uk/Spm/Software/Spm12/, https://Bio.Tools/SPM) software and the Clusterize toolbox. This process, detailed in prior studies (Clas et al. 2012; de Haan et al. 2015), involved marking lesions and using a clinical tool for normalizing the images based on an older population template (Rorden et al. 2012). For the lower quality images, a manual approach was taken. These images were recreated using the T1 single-subject template from the Montreal Neurological Institute, employing MRIcron software (Rorden and Brett 2000), following methods outlined in earlier research (Manuel et al. 2013; Chouiter et al. 2016).

### Connectome-based Lesions-symptom Mapping Analyses

#### Disconnection Severity

We conducted the CLSM analyses using the lesion Quantification Toolbox to model the impact of lesions on the structural brain connectome with the procedure by Griffis et al. 2021. The toolbox models the intersection between lesion masks and the selected structural tractography atlas generating the parcel-wise disconnection severity, the selected parcel-wise atlas is combined with the tractography atlas to model a structural connectome featuring all anatomically plausible direct connections between pairs of gray matter regions of interest (ROIs). This value estimates the severity of lesion-induced structural disconnections of each ROI-ROI direct connection, and is expressed as the percentage of reduction of streamlines composing the ROI-ROI connection.

We conducted these analyses using an “End” connection criterion with the deterministic HCP842 tractography template Yeh et al. 2018 and the fMRI-based parcels atlas from Schaefer et al. 2018, featuring 135 cortical and subcortical gray matter areas.

#### Relation Between White Matter Disconnections and Variables of Interest

We investigated the association between damage to white matter tracts and whole-brain direct ROI-ROI structural connections using the publicly available Matlab script used in Sperber et al. 2022 (adapted version and link to the original script are available on the publicly accessible digital repository “Zenodo” (10.5281/zenodo.7845740). The script computes a general linear model between the specific disconnection severity measure (parcel-wise disconnection severities) and a single variable of interest with a mass-univariate approach (ROI-ROI pair is independently tested) and controls for family-wise error rate (FWER) using a “maximal statistic permutation” correction of significance threshold (Nichols and Holmes 2002).

**Fig. 2:**
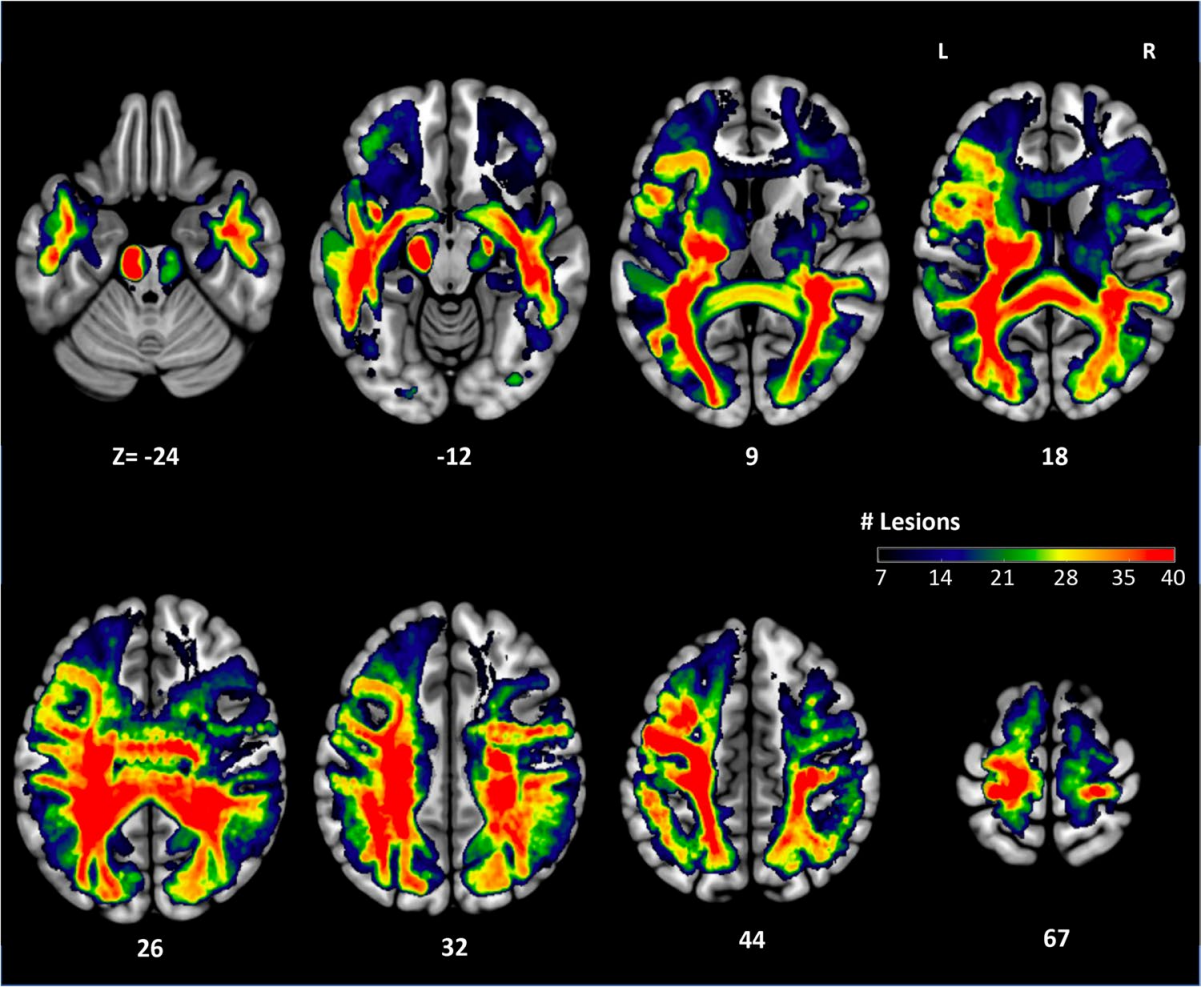
Coverage map of the voxel-wise disconnections for voxels with disconnection in at least seven patients: The color of each voxel represents the number of patients whose lesions produced a reduction of streamline density passing through the voxel. MNI z coordinates of the axial sections are reported

We tested the impact of parcel-wise direct disconnections on the performance index, the N2 and P3 GFP peaks, and assuming that lesions would cause a reduction of the three variables, we ran the analyses one-tailed with a corrected alpha of 0.05 based on 5000 permutations. We analyzed only ROI-ROI connections that were damaged in at least 7 and 10 patients, respectively. The number was chosen to balance between statistical power and sufficient brain coverage, resulting in a total 652 ROI-ROI connections being analyzed (see Fig. 2).

To generate the coverage map, the volume of the HCP-842 streamline tractography atlas is divided into 1mm^3^ voxels, resulting in a variable number of streamlines passing through each voxel with different directions. Each participant's 3D lesion mask is embedded in the tractography atlas modeling the disconnection of the streamlines intersected by the lesion. All voxels laying on the entire path of each single disconnected streamline are then considered as disconnected by the lesion. In other words, the disconnection of one or more of the streamlines passing through a voxel would cause the voxel to be considered as disconnected by the lesion. As a result, the 3D coverage map in Fig. 2 represents for each voxel how frequently streamlines passing through the voxel were disconnected by lesions in the cohort.

## Results

### Behavior

In the Go/No-Go task, the average rate of false alarms (commissions errors) was 15 ± 8.5% (mean ± SD) and the average reaction time for a correct Go response was 426.2 ± 73.4 ms. The percentage of hits (Hit too late) exceeding the threshold was 56 ± 11.8% and those below the threshold was 44 ± 11.8%. In addition, the average of d' and c across patients was 3.14 ± 0.67 and -0.48 ± 0.28, respectively. This result indicate that the task stimuli were easily discriminable and that the inhibition cognitive component was properly loaded.

### Connectome-based Lesion-symptom Mapping

#### Disconnections Reducing Inhibitory Control Performance

The left arcuate fasciculus and left thalamocortical pathway were critical for IC performance We also found a role for the left intrahemispheric (fronto-temporal, fronto-basal) and for the interhemispheric connections between left temporoparietal and right temporal areas (α = 0.01).

#### Disconnections Reducing the N2 GFP

**Fig. 3:**
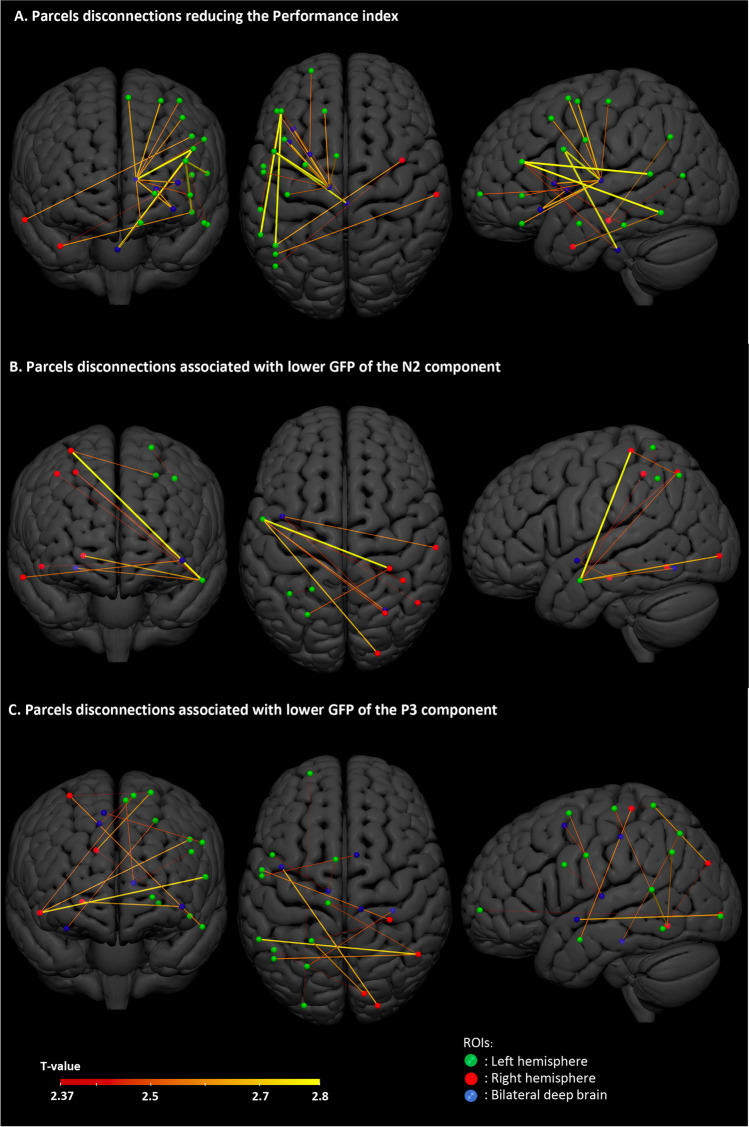
Parcel-wise disconnections impairing Go/NoGo performance: Lines represent the ROI- ROI disconnections associated with lower values of the explored variable at p < 0.01; Diameter and color are proportional to the strength of the linear relationship; **A** disconnections reducing the performance index, Blue nodes correspond to the left thalamus, left Putamen, left Anterior Insula (2 nodes) and Brainstem; **B** Disconnections reducing the GFP of the N2 component, Blue nodes correspond to the left Posterior Insula and right fusiform gyrus; **C** Disconnections reducing the GFP of the P3 component, Blue nodes correspond to the left thalamus, left posterior Insula, right fusiform gyrus, right middle Cingulate gyrus and right supplementary motor (2 nodes)

We found an influence on the N2 component of disconnection between the left temporal and right superior parietal regions, as well as for interhemispheric connections between the left temporal and right parieto-occipital regions, between the left insula and right temporal and parietal areas as well as an interhemispheric parieto-parietal connection (α = 0.01; Fig. 3B).

#### Disconnections Reducing the P3 GFP

We found a role in the P3 component for interhemispheric connections between left temporal and parietal areas and right temporal and occipital areas. Minor connections were also found between left sensorimotor areas and right cingulum/motor supplementary area, in addition to left intrahemispheric fronto-basal connections (α = 0.01; Fig. 3C).

## Discussion

We combined connectome-based lesion-symptom mapping approach (CLSM) with behavioral and electrophysiological indices of motor inhibitory control (IC) to investigate in a sample of 96 first-time stroke patients whether and how disruptions in anatomic connectivity influence performance and brain functional activity. We focused on two key functional executive control processing phases, namely the detection of conflict between task demands and response tendencies (as indexed by the N2 ERP component) and the implementation of inhibition commands (P3 component). The CLSM analysis revealed a role of left frontotemporal and frontobasal intrahemispheric connections, as well as interhemispheric connections between the left temporoparietal and right temporal areas in Go/NoGo performance. Moreover, we found a role of the connections between the left temporal and right superior parietal area for the conflict N2 component and between the left temporoparietal area and right temporal and occipital area for the inhibition P3 component.

Our results for a role of the left arcuate fasciculus and left corticothalamic pathway tracts in Go/NoGo performance corroborate previous reports for a role of these areas in executive control, especially in the language domain (Rilling et al. 2008; Anziano et al. 2023). They however point to a prominent role of left-lateralized anteroposterior communication, which may follow from the difficulty experienced by the patient to complete speeded executive tasks. The left ventrolateral prefrontal cortex has indeed been suggested to support right-lateralized frontal structure with aging and when executive tasks become more demanding (Kang et al. 2022). This could be the case in our sample, as stroke patients are typically more fatigable and show difficulty in maintaining executive performance.

The electrophysiological results of the N2 ERP component analysis do not confirm our hypothesis of a role for direct connections with the anterior cingulate in conflict detection (Braver et al. 2001), but that interhemispheric communication between the left temporal and right superior parietal is relevant for this functional processing step. The left temporal area has been involved in language processing (Maess et al. 2006), while the right superior parietal region in bottom-up attention and stimulus-response conflict resolution (Corbetta and Shulman 2002). The communication between these areas thus appears crucial in this processing step, most likely due to their role in identifying the letter-based response condition and allocating attentional resources to the demand for executive control.

Likewise, our CLSM result for the P3 inhibition-related component does not confirm a role for direct connections with the right inferior frontal gyrus in global motor inhibition (Aron et al. 2014), but rather suggests that interhemispheric communication between the left temporal parietal area and right temporal and occipital areas is necessary for this functional processing step (Vidal et al. 2012; Fine et al. 2020). These areas have been previously shown to be involved in motor inhibition and over the same time periods as our ERP components of interest (Liddle et al. 2001; Vidal et al. 2012); in implementing stimulus–response mapping rules (Manuel et al. 2010); attentional reorienting under response inhibition (Ko et al. 2016) and attentional control (Murphy et al. 2020; Fine et al. 2020).

Swick and Chatham (2014) challenge Aron et al. (2014) theory for a prominent role of the right inferior frontal cortex (rIFC) as the main “braking” area in inhibitory response control, notably by pointing that left IFC (virtual) lesions also impair inhibition, the unspecific role of the IFC in inhibition and the potential confounding effects of context monitoring or saliency detection versus actual braking. In these debates, our findings concur with the fact that plastic reorganization must be taken into account in the study of post-lesional measures of response inhibition, and for a role of interhemispheric and left-lateralized white matter tracts in this function. Accordingly, and with regard to the view by Hampshire and Sharp (2015), we would further note that our results speak in favor of the view that inhibitory control emerges from the dynamic interactions of domain-general frontoparietal networks. These networks modulate local lateral inhibition processes throughout the cortex, supporting a wide range of cognitive functions beyond just inhibition. As suggested by our results, flexible, broader neural networks are critical for inhibitory control, rather than prominent role of localized cortical areas.

The involvement of more posterior and left-lateralized regions we found might also be attributed to the influence of attentional resources on the IC. We did not directly control for coexisting deficits across other cognitive domains, and attentional impairments are frequent in stroke patients. Therefore, reduced attention may not only have impacted performance, but may also have impaired the neurophysiological processes more directly related to IC, as indexed by the association of N2 and P3 components with disconnection of temporal and parietal regions typically involved in attentional functions. This may result from the complexity of executive functioning and the interdependence between its cognitive subcomponents. In addition, while CLSM is less sensitive to sample size than voxel-based lesion symptom mapping (Anziano et al. 2023), the heterogeneous distribution of statistical power inherent to brain-damaged patients samples may have resulted in false negatives, especially in prefrontal areas where an asymmetry disfavoring the right hemisphere was also present. The absence of involvement of prefrontal tracts should thus be interpreted with caution. Further studies with more largely distributed lesions should thus be conducted to confirm our first-pass utilization of this original combination of methods to confirm and extend our results.

Overall, our results provide the first demonstration that combining lesion-symptom mapping approaches with functional indices of cognitive processes can shed new light on the role of anatomic connectivity in executive performance and functional activity. Most notably, it may help refine the interpretation of classical electrophysiological markers of executive control in stroke patients.

## Data Availability

Patients have neither given their consent to share their coded data on a public repository nor to anonymize their data for such purpose, as confirmed by the Ethical Committee of Vaud (CER-VD). The President of the CER-VD (secretariat.cer@vd.ch), is ready to answer any query regarding this issue (please mention the study number 2016–00517) in each related correspondence to the CER-VD).
